# Language outcomes from the UK-CDI Project: can risk factors, vocabulary skills and gesture scores in infancy predict later language disorders or concern for language development?

**DOI:** 10.3389/fpsyg.2023.1167810

**Published:** 2023-06-16

**Authors:** Lana S. Jago, Katie Alcock, Kerstin Meints, Julian M. Pine, Caroline F. Rowland

**Affiliations:** ^1^Department of Psychology, University of Liverpool, Liverpool, United Kingdom; ^2^Department of Psychology, Lancaster University, Lancaster, United Kingdom; ^3^School of Psychology, University of Lincoln, Lincoln, United Kingdom; ^4^Language Development Department, Max Planck Institute for Psycholinguistics, Nijmegen, Netherlands; ^5^Donders Institute for Brain, Cognition and Behaviour, Radboud University, Radboud, Netherlands

**Keywords:** vocabulary, CDI, health risk factors, demographic risk factors, language development, language impairment, language disorder

## Abstract

At the group level, children exposed to certain health and demographic risk factors, and who have delayed language in early childhood are, more likely to have language problems later in childhood. However, it is unclear whether we can use these risk factors to predict whether an individual child is likely to develop problems with language (e.g., be diagnosed with a developmental language disorder). We tested this in a sample of 146 children who took part in the UK-CDI norming project. When the children were 15–18 months old, 1,210 British parents completed: (a) the UK-CDI (a detailed assessment of vocabulary and gesture use) and (b) the Family Questionnaire (questions about health and demographic risk factors). When the children were between 4 and 6  years, 146 of the same parents completed a short questionnaire that assessed (a) whether children had been diagnosed with a disability that was likely to affect language proficiency (e.g., developmental disability, language disorder, hearing impairment), but (b) also yielded a broader measure: whether the child’s language had raised any concern, either by a parent or professional. Discriminant function analyses were used to assess whether we could use different combinations of 10 risk factors, together with early vocabulary and gesture scores, to identify children (a) who had developed a language-related disability by the age of 4–6 years (20 children, 13.70% of the sample) or (b) for whom concern about language had been expressed (49 children; 33.56%). The overall accuracy of the models, and the specificity scores were high, indicating that the measures correctly identified those children without a language-related disability and whose language was not of concern. However, sensitivity scores were low, indicating that the models could not identify those children who were diagnosed with a language-related disability or whose language was of concern. Several exploratory analyses were carried out to analyse these results further. Overall, the results suggest that it is difficult to use parent reports of early risk factors and language in the first 2 years of life to predict which children are likely to be diagnosed with a language-related disability. Possible reasons for this are discussed.

## 1. Introduction

Young children with early delays in language acquisition are at an increased risk for developing persisting language impairments (Rice et al., 2008). As a result, policy-makers and lobby groups often recommend that the language development of at risk children is monitored through their first years, and that they receive targeted support aimed at improving their communication environments (All Party Parliamentary Group on Speech and Language Difficulties, 2013; Save the Children, 2015).

However, though it is widely accepted that children’s early vocabulary and gesture use correlates with their later language ability at the group level, at least in children within the typical range (Westerlund et al., 2006; Henrichs et al., 2011; Rescorla, 2011), the role of early language in *predicting* later language-related disability in individuals with sufficient accuracy is less clear cut. Many children who experience an early delay in language acquisition are later diagnosed with a developmental language disorder, but many also catch up with their peers before they enter school (Grossheinrich et al., 2019). Therefore, there is not a straightforward, linear, relationship between early and later language. This makes it very difficult to identify predictors of, for example, later language disorder (Dale et al., 2003; Westerlund et al., 2006). In particular, Westerlund et al. (2006) reported that a measure of vocabulary size at 18 months was not sensitive enough to identify children with language impairment at 3 years. The authors conclude that, although early language skills were associated with later language skills, alone, they were not sensitive enough to identify children with language impairment.

Exposure to a number of adverse health and demographic risk factors is also associated with delays in language development (Paul, 2000; Campbell et al., 2003; Reilly et al., 2010). However, once again, while a number of risk factors predict later language outcomes on a group level (Reilly et al., 2007, 2010), it is not clear that they accurately predict later language proficiency on an individual level. This means that we are currently unable to identify which children are likely to develop problems in their language acquisition using only exposure to risk factors as a predictor (Roos and Ellis Weismer, 2008; Bishop et al., 2012; Duff et al., 2015).

It is, however, possible that the accuracy of our predictive models could be improved by combining detailed measures of early language abilities (vocabulary and gesture use) with health and demographic risk factors (e.g., combining productive vocabulary with measures of receptive vocabulary, gestures, risk factors and early concern for language impairment). Thus, the goal of the present paper was to determine if parental assessments of vocabulary and gesture use in infancy, together with health and demographic risk factors, could be used to predict whether a child was later (by the age of 4–6 years) diagnosed with a disability that was likely to affect spoken English language proficiency (e.g., developmental disability, language disorder, hearing impairment). Because we recognized that some 4-6-year-olds may not yet have received a diagnosis, we also tested the effect of our risk factors on later language using a broader, more inclusive, measure; whether, by the age of 4–6 years, the child’s language had raised any concern, either by a parent or professional. By including both measures we can gain a fuller picture of the link between early risk factors and later susceptibility to a language-related disability. In the remainder of this introduction, we review the relevant research on risk factors, before outlining the current study.

### 1.1. Health risk factors

A number of health factors have been shown to be robustly associated with language in young children; in particular child sex/gender^1^, prematurity, low birth weight, ear infections, family history of speech or language impairments, and associated (non-language) developmental disabilities (Huttenlocher et al., 1991; Marschik et al., 2007; Barre et al., 2011; Kenyhercz and Nagy, 2022). For example, Reilly et al. (2010) found that multiple health-related risk factors (child sex/gender, low birth weight and family history of speech and language difficulties) predicted variance in language skills at age 4 years. Similarly, Jansson-Verkasalo et al. (2004) reported that being born premature was associated with poorer performance on measures of comprehension at 2 years.

However, such research often yields weak effects (Harrison and McLeod, 2010; Dale and Hayiou-Thomas, 2013; Fisher, 2017; Jin et al., 2020). This means that though the risk factor is correlated with language, it cannot be used to classify children as having a language delay or disorder. For example, Harrison and McLeod (2010) found that while health risk factors correctly classified children who were not attending speech-language pathology services, they only correctly classified 2.6% of children who were attending these services, showing that health risk factors were not sensitive enough to identify children with language impairment.

### 1.2. Demographic risk factors

Two demographic factors that have been shown to robustly predict language development over time are maternal education and household income (Reilly et al., 2010; Fisher, 2017). It is likely that these factors impact language development together because they interact; rate of household income can be dependent on parental education and together they determine socioeconomic status (SES). Previous research has suggested that the role of maternal education on language development may act *via* the mother’s own language skills; parents with higher educational attainment have more advanced linguistic skills and are therefore more likely to produce rich linguistic input from which children can learn (Rowe et al., 2009; Rowe, 2018; Vernon-Feagans et al., 2020) although there is also a genetic component making it hard to disentangle cause and effect (Kovas et al., 2005). In addition, there are other factors at play; parents suffering from living in chaotic and crowded conditions will have less opportunity to engage in long periods of linguistic interaction with their children (Evans et al., 1999; Rowe, 2018; Fan et al., 2021). However, again, an association is not enough for accurate classification. For example, Harrison and McLeod (2010) found family income did not predict whether or not children were attending speech-language pathology services, and also reported that the effect of maternal education on predicting later language is weak (Harrison and McLeod, 2010).

### 1.3. Early concern about language development

Parental concern for language development is an often neglected, but promising factor, since it has been shown to support the identification of developmental delays and language impairment (Glascoe, 1991; Sim et al., 2019; Wallisch et al., 2020). A delay in vocabulary development is one of the reasons that parents first seek support for their children’s development (Rescorla, 2011; Solgi et al., 2022). When combined with clinical observations, parental evaluation of their children’s language development increases the accuracy with which paediatricians can detect developmental complications (Glascoe et al., 1989; Glascoe and Dworkin, 1995). Therefore, it may be possible to use parental concern, in combination with other risk factors, to support the detection of delays in language development, before language impairment is identified by a clinician or therapist.

The research outlined above shows that although multiple studies have identified associations between early risk factors and language, they have not concluded that it is possible to use these factors to identify, in individual children, the likelihood of later language-related disabilities such as developmental language disorder [DLD, previously called specivic language impairment (SLI)]. A plausible explanation is that the size of the effect of these risk factors in language acquisition are quite weak overall. In support of this idea is the finding that where effects are found, effect sizes are often small (Stolt et al., 2009; Carroll and Breadmore, 2017), and that studies with larger sample sizes are often more successful at detecting relationships between risk factors and language acquisition (Kennedy et al., 2006; Winskel, 2006; Barre et al., 2011; Van Noort-Van Der Spek et al., 2012).

If the effects are weak (i.e., effect sizes are small), this might explain why some measures discriminate well between faster learners, or learners in general (i.e., correlate with language), but have very little predictive power when identifying which individual children will develop a language-related disability. For example, boys tend to be somewhat slower word learners than girls on average (Eriksson et al., 2012) but the differences are so small, and the overlap in standard deviation so large, that sex/gender has very little discriminatory power on the level of the individual. In particular, Reilly et al. (2010) found that nine risk factors were successful in predicting individual differences in language scores at 4 years, but only three of them (family history of speech or language problems, low maternal education and SES) allowed the authors to discriminate between children with and without expressive DLD.

If the effects are weak, then, although risk factors when examined in isolation may not be strong enough to predict language impairment, they might gain more substantial predictive power when combined. Using multiple risk factors, combined with measures of earlier language skills, might then increase the chances of successfully predicting later language impairment. However, very little research has investigated how earlier language skills and multiple risk factors might interact to increase the risk of developing persistent language impairments. If we can show that a combination of risk factors and language skills identified early can predict later language impairment, we can use these factors as a starting point for informing early intervention based on risk.

### 1.4. The current study

The current study had three aims. First, we investigated whether, using a combination of risk factors and early vocabulary and gesture scores at time 1 (15–18 months), we could predict which children would go onto develop a language-related disorder at time 2 (4–6 years). We identified children with language-related disorder at age 4–6 years in two different ways. In our first category (Identified Disability group), we included only those children who had been diagnosed with a disability that was likely to affect spoken English proficiency by the age of 4–6 years (e.g., developmental disability, language disorder or hearing impairment). However, since many children do not have a diagnosis by age 4–6 years, we also created a broader category that included both children with an identified disability and children for whom a parent or a professional had expressed ‘concern’ about their language development (Overall Concern group). This second category is based on the premise that some children with a disability are not yet diagnosed at 4–6 years in the UK, and that the concern of a parent/professional may be a good indicator of an, as yet, undiagnosed language-related disability (Glascoe et al., 1989; Glascoe and Dworkin, 1995).

Our second aim was to determine whether there was continuity in concern over time; whether overall concern for language development at 4–6 years was predicted by parental concern for their child’s language development between 15 and 18 months. We anticipated that parents’ concerns about their children’s language development in late infancy would predict later overall concern for children’s later language development.

Our third and final aim was to investigate if risk factors and early language skills together could predict whether or not children’s language development caught up *after* concern for their language development had been registered. We anticipated that children with bigger vocabularies and higher gesture scores at time 1, as well as those who experienced fewer risk factors, would be more likely to catch up compared to children experiencing more risk factors and with lower vocabulary and gesture scores.

## 2. Methods

### 2.1. Participants

The cohort were a subset of the 1,210 parents who had taken part in the UK-CDI Project when their children were between 15 and 18 months. The UK-CDI Project collected parental report data from across the United Kingdom to establish norms for productive and receptive vocabulary, and gestures for children from 8 to 18 months. Parents completed a vocabulary and gesture questionnaire (a Communicative Development Inventory, or CDI; Alcock et al., 2020), and a family questionnaire containing questions about child health, familial risk of language and literacy disorders, and demographic characteristics. The original cohort were representative of the UK population as a whole in terms of a range of demographic factors (e.g., socio-economic status, sex/gender, region, nation, marital status etc).

We sent out follow-up questionnaires (see design section below) to all of the families from the original cohort who had agreed to be contacted for further studies, had provided contact details, and whose children were between 15 and 18 months when they completed the UK-CDI (n = 370; 78 of whom had productive vocabulary scores in the bottom 25th percentile). We received 147 (40%) responses. One family was excluded because their response was incomplete, so the final sample size was 146.

The mean age of the 146 children included in the follow-up project was 16 months 25 days (age range: 15 months, 3 days −18 months, 28 days) during the UK-CDI Project (time 1), and 5 years and 3 months (age range: 4 years, 3 months–6 years, 4 months) at follow-up (time 2). All children were monolingual English learners. Table 1 shows the number of children in the different risk factor categories at time 1 and Table 2 shows the number of children in each quartile for vocabulary and gesture use at time 1 (Note that there were differences in the maternal education and household income of the parents who did/did not take part in the follow-up study, but not in any of the other risk factors; see Supplementary materials at https://osf.io/gvz3x/).

**Table 1:** Number of children with and without each risk factor at time 1.

	Female			Male		
Risk factor	*n* with the risk factor	*n* without the risk factor	Missing data	*n* with the risk factor	*n* without the risk factor	Missing data
Health problems at time 1 (total)						
Prematurity time 1	5	65	0	8	68	0
Low birth weight time 1	6	64	0	5	71	0
Ear infection at time 1	1	69	0	2	74	0
Familial risk (someone in family) time 1	12	58	0	12	63	1
Developmental disability time 1	0	69	1	0	76	0
Visual or hearing impairment time 1	1	69	0	1	73	2
Language concerns at time 1						
Hearing or communication concerns at time 1		2	68	0	8	68
Demographic factors at time 1 (total)						
Maternal education time 1	9	61	0	3	73	0
Household income time 1	18	52	0	16	60	0
Language catch-up after concern at time 1	1	9	0	10	17	0

For this table, language not catching up after Concern at time 1 is categorized as ‘with the risk factor’ and language catching up is categorized as ‘without the risk factor’.

**Table 2:** Number of children in each quartile for vocabulary and gestures at time 1 (group membership).

Variable	0–25th	25–50th	50–75th	75–100th
Productive vocabulary	39	30	39	38
Receptive vocabulary	33	32	32	49
Gestures	30	36	43	37

### 2.2. Design

We used a parent-report questionnaire to follow up children who previously took part in the UK-CDI Project, and had agreed to be contacted for future studies. This study was granted ethical approval by The University of Liverpool’s Research Ethics Subcommittee for Non-Invasive Procedures for the study Language Development in Late Talkers (Institute Review Board protocol number: RETH000764). Predictor variables were measures that were derived from the answers given at time 1. These were: receptive vocabulary quartile, productive vocabulary quartile and gesture quartile, child sex/gender, prematurity, low birth weight, ear infections at time 1, familial risk for speech or language impairment, ear infection lasting more than 6 months, developmental disability, maternal education, household income, hearing or communication concerns at time 1. Outcome measures were derived from answers at time 2: Identified Disability, Overall Concern, Catch up. (See below for information about how these measures were derived).

### 2.3. Sampling and data collection procedures

The first data collection phase (time 1) took place as part of the UK CDI Project from 2013 to 2015. Details of how time 1 data were collected can be found in Alcock et al., 2020. The data for the follow-up phase (time 2) was collected between 2017 and 2018. We used the database from the UK-CDI Project to follow-up families who had consented to be re-contacted for future research. Parents were given a £5 shopping voucher for completing the follow-up questionnaire.

### 2.4. Measures and procedure

#### 2.4.1. The UK-CDI

Communicative Development Inventories (CDI) are parent report checklists of the words, gestures and sentences that young children understand and say. Parents complete these questionnaires by indicating if their child uses or understands the words, sentences and gestures listed. The UK-CDI Words and Gestures is standardized for the UK population for vocabulary and gesture scores in children aged 8–18 months and has good validity and reliability (see Alcock et al., 2020; total possible scores are 395 for vocabulary and 63 for gesture).

#### 2.4.2. The family questionnaire

The family questionnaire asks a range of questions about a child’s health and family background. This questionnaire was designed for the UK-CDI Project (for details of construction, see Alcock et al., 2020) and was used to collect information about demographic and health risk factors, including prematurity, birth weight, family history of language delay or dyslexia, and SES.

#### 2.4.3. Follow-up questionnaire

The follow-up questionnaire was used to investigate the later language outcomes of the children who took part in the UK-CDI project. The key questions for this study were those that asked about parental concern for language development, details of those concern (if any), whether the Healthy Child Programme’s 2 Year Review identified a delay in language development, whether the children had been diagnosed with a developmental disability or language disorder, and whether the children had a visual or hearing impairment. The Healthy Child Programme 2 Year Review (Department of Health, 2009) is part of the *Healthy Child Programme: Pregnancy and the first five years of life*, which is run in England and Wales. This review is designed to optimize child development by reviewing all children in England and Wales between 2 and 2 years, 6 months.

At time 2, parents who had provided an email address at time 1 were sent an email containing a link to complete the questionnaire online. For parents who only provided a home address, a paper copy of the questionnaire was sent out with a prepaid return envelope included. See the Supplementary materials at https://osf.io/gvz3x/ for a copy of this questionnaire.

### 2.5. Data coding

#### 2.5.1. Risk factor scores at time 1

Ten questions probed the child’s susceptibility to 10 risk factors. All information was collected at time 1 when children were 15–18 months old. Answers to risk factor questions were scored as 1 if the child had the risk factor, and 0 if they did not

1. Concern: answering yes to “have you or anyone else had any concerns about your child’s hearing or communication?” = score of 1

Physical/health factors:

1. Child sex/gender: operationalized as a binary variable (male female); male = score of 1
2. Prematurity: operationalized as a binary variable (premature/not premature): born before week 36 = score of 1
3. Low birth weight: operationalized as a binary variable (low/not low); weighing less than 5 lb. 8 oz. when born = score of 1
4. Ear infection: operationalized as answering yes to the question “has your child had an ear infection/ glue ear for longer than 3 months, 4 to 6 ear infections within a 6 month period, or another identified hearing problem?” = score of 1
5. Familial risk of language/literacy disorder: answering yes to “is there anyone in the immediate family with speech/language difficulty or dyslexia?” = score of 1
6. Developmental disability: answering yes to “does your child have a developmental disability?” = score of 1
7. Hearing or visual impairment: answering yes to “does your child have a hearing or visual impairment?” = score of 1

Demographic factors:

1. Maternal education: selecting “no formal qualifications” or “GCSE/O level/NVQ level 1 or 2” = score of 1
2. Household income: selecting “£0–£14,000” or “£14,000–£24,000” = score of 1

Although many studies look only at maternal education, we used both education and income categories as there is evidence that different operationalisations of SES can have different effects (see, e.g., De Cat, 2021). The cut off scores for maternal education and household income used above were designed to determine low SES status. For household income, families with income of around £22,800 per year were considered to have low income at the time of data collection (Department for Work and Pensions, 2019). Low maternal education was established as having no formal qualifications or GCSE/O level/ NBQ level 1 or 2. Previous research has shown that children of mothers with fewer than 12 years of education are at increased risk for persisting language impairment (Stanton-Chapman et al., 2002).

### 2.6. Language measures at time 1

#### 2.6.1. Division by quartiles: group membership

Because we wanted to identify whether being in the bottom 25th percentile for vocabulary/gesture at time 1 would predict language-related disability at time 2, we divided the children into four groups based on vocabulary and gesture scores between 15 and 18 months using the UK-CDI norms. The UK-CDI norms were created using the entire UK-CDI Project sample and provide percentile cut-offs for productive and receptive vocabulary, and for gestures, for each month. Children were split into four groups based on percentiles: 0–25th, 25–50th, 50–75th, and above 75th percentiles. Each child was placed into one of the four groups separately for productive vocabulary, for receptive vocabulary and for gesture use.

#### 2.6.2. Concern scores at time 2

Five questions asked about the child’s likelihood of having a language-related disorder at time 2:

1. Parental concern for language development: answering yes to “have you ever worried that your child’s speech was delayed compared to other children the same age?” = score of 1
2. Developmental disability: answering yes to “does your child have a developmental disability?” = score of 1
3. Diagnosis of language disorder: answering yes to “has your child been diagnosed with any of the following language disorders?” = score of 1
4. Hearing or visual impairment: answering yes to “does your child have a hearing or visual impairment?” = score of 1
5. Professional concern for language development. Identification by the Healthy Child Programme’s 2 Year Review (the Two Year Check): answering yes to “did this programme identify any delays with your child’s speech, language or communication abilities?” = score of 1

The parents’ answers to these questions were used to calculate two overall scores for each child:

- Identified Disability: Children whose parents answered yes to any one of questions 2, 3 or 4 above were given a score of 1. Children whose parents answered no to questions 2, 3 and 4 were given a score of 0.
- Overall Concern: Children whose parents answered yes to any of the question above were given a concern score of 1. Those whose parents answered no to all of the questions above were given a concern score of 0.

Twenty children (13.70%) fit the criteria for having an Identified Disability (developmental disability =10, language disorder =1 and/or visual or hearing impairment = 12). Forty-nine children (33.56%) were identified as having language that was of Overall Concern at time 2. Note that DLD is estimated to affect approximately 7.58% of the population (Norbury et al., 2016).

Parents who answered yes to the question “have you ever worried that your child’s speech was delayed compared to other children the same age?” were also asked a catch-up question (“Did your child’s speech eventually catch up with that of other children the same age?”). Of the 37 parents who answered this question, 26 reported that their children’s language had caught up with children the same age, and 11 reported that it had not caught up (7.53% of the full sample of 146 children).

### 2.7. Analysis strategy

All analyses were conducted using one-tailed tests, as all hypotheses are unidirectional hypotheses. All outliers were included, unless it was determined that the data point was due to experimenter or participant error. Chi^2^ analyses were performed in R (R Core Team, 2017, R version 3.4.1) using R Studio (Version 1.0.153) using the CrossTable function as part of the gmodels package (Warnes et al., 2018). Logistic regressions were performed in R using the glm function as part of the pscl package (Jackman, 2010). Discriminant function analyses were run in SPSS Statistics 24.

Discriminant function analysis is a statistical technique used to determine how well predictor variables discriminate between two or more naturally occurring groups. Here we used it to determine which different combinations of risk factors gave us the best classification accuracy of children into our two outcome groups. For the first set of analyses the two groups were: children with an Identified Disability (1) and children without an Identified Disability (0) at time 2. For the second set of analyses the two groups were: children for whom concern about language had been expressed (1) and children for whom concern about language had not been expressed (0) by time 2. Discriminant function analysis yields an overall accuracy figure (how well the model performs at discrimination overall), and sensitivity and specificity values. The sensitivity value measures the ability of the model to correctly classify children who have an identified disability or for whom there is concern for language development (true positives). Specificity measures the ability of the model to correctly classify children who do not have an identified disability or for whom there has been no concern expressed about their language development (true negatives).

Sensitivity and specificity rates between 70 and 80% are deemed acceptable for diagnostic assessments (e.g., for autistic spectrum disorder; Bright Futures Steering Committee and Medical Home Initiatives for Children with Special Needs Project Advisory Committee, 2006). Therefore, we consider accuracy, sensitivity and specificity and values above 70% to be adequate in the present analyses. Discriminant function analyses also provides standardized canonical coefficients for each variable. These coefficients allow us to compare the weighted importance of each variable in predicting group membership.

## 3. Results

### 3.1. Descriptive statistics

Table 1 shows the number of children in the different risk factor categories at time 1 (15–18 months) and Table 2 details the number of children in each quartile group for productive vocabulary, receptive vocabulary and gestures at time 1 (15–18 months). Table 3 shows the number of children who had been diagnosed with an identified disability and/or whose language had raised concern at time 2.

**Table 3:** Number of children with and without each risk factor at time 2.

		Female			Male		
Risk factor		*n* with the risk factor	*n* without the risk factor	Missing data	*n* with the risk factor	*n* without the risk factor	Missing data
Identified Disability (answering “yes” to at least one of the three questions on diagnosis of developmental disability, diagnosis of language impairment, having a visual or hearing impairment at time 2)		4	66	0	16	60	0
Overall Concern at time 2 (answering “yes” to any of the 5 questions which denote concern)		16	54	0	33	43	0
Concern expressed by parent at time 2	10	60	0	27	49	0
	Concern expressed at 2 Year Review time 2	6	56	8	16	45	15
	Diagnosis of developmental disability time 2	3	67	0	7	69	0
	Visual or hearing impairment time 2	1	69	0	11	65	0
	Diagnosis of language disorder time 2	0	70	0	1	75	0

The total number of children with any one risk factor at time 2 may exceed the total number of children with overall concern at time 2. This is because some children experienced more than one risk factor.

We checked for collinearity in our predictor variables as this can affect the interpretability of regression models. We ran Chi^2^ analyses between each pair of variables to check for associations, and then, for any two variables that yielded significant Chi^2^ scores, we followed this up with a Cramer’s V post-test to determine collinearity. Cramer’s V provides an effect size where values vary between 0 and 1, with 0 indicating no collinearity and 1 indicating high collinearity. The Chi^2^ analyses revealed that eight of the predictor variables were significantly associated (prematurity and low birth weight; low birth weight and family history of language delay or dyslexia; ear infection at time 1 and visual or hearing impairment at time 1; family history of language delay or dyslexia and a visual or hearing impairment at time 1; family history of language delay or dyslexia and maternal education; visual or hearing impairment at time 1 and maternal education; maternal education and household income; productive vocabulary group and receptive vocabulary group; productive vocabulary and gesture group; receptive vocabulary and gesture group). However, none of these variables were highly collinear (all Cramer’s V values below 0.70). Collinearity between developmental disability at time 1 and other variables could not be established because no parents reported developmental disability at time 1.

### 3.2. Predicting identified disability at time 2 from risk factors and language at time 1

First, we ran discriminant function analyses to assess the discriminatory ability of the risk factors and language group at time 1 to correctly classify children into two groups: Identified Disability or No Identified Disability.

We ran five analyses (see Table 4 for the overall results, and Table 5 for the standardized canonical coefficients for each variable in each model, which indicate the weighted importance of each variable in predicting group membership). The first analysis included only language group at time 1 (quartile groups for productive vocabulary, receptive vocabulary and gestures at time 1) to determine if this could predict group membership at time 2 (Identified Disability, No Identified Disability). This model failed to correctly classify children into their groups, *r* = 0.13, *χ*^2^ = 2.36, *df* = 3, *p* < 0.50. The accuracy of the model was high, 86.30%, and it had good specificity (100.00%), meaning that it did well in classifying children in with no identified disability. However, the sensitivity was poor at 0.00%, so the model did not do well in identifying the children in the Identified Disability group at time 2.

**Table 4:** Results from the discriminant function analyses distinguishing between children with and without an identified disability at time 2.

Variable	*r*	*χ*2	*n*	df	*p*	Accuracy	Sensitivity	Specificity
Language group at time 1 (quartile groups for vocabulary and gesture scores at time 1)	0.13	2.36	146	3	0.50	86.30%	0.00%	100.00%
Health factors (sex/gender, prematurity, low birth weight, ear infection, visual or hearing impairment, family history, developmental disability)	0.35	17.90	143	6	0.006	86.80%	10.50%	98.40%
Demographic factors (maternal education, family income)	0.16	3.62	146	2	0.16	86.30%	0.00%	100.00%
Concern at time 1	0.13	2.39	146	1	0.12	86.30%	0.00%	100.00%
All variables	0.40	22.87	143	12	0.03	86.10%	15.80%	96.80%

**Table 5:** Standardized canonical discriminant function coefficients of each discriminant function analysis predicting overall and identified concern.

Model	Variable	*r* (Overall Concern)	*r* (Identified Concern)
Language group	Productive vocabulary group	1.07	0.66
	Receptive vocabulary group	−0.18	−0.36
	Gesture group	0.08	0.79
Health factors	Sex/Gender	0.69	0.67
	Prematurity	−0.25	−0.64
	Low Birth Weight	0.45	0.68
	Ear Infection	0.70	0.60
	Family history	0.22	−0.11
	Visual or hearing impairment	−0.15	0.16
Demographic factors	Maternal education	−0.48	−0.09
	Household income	1.02	1.02
Concern at time 1	Concern at time 1	1.00	1.00
All variables	Sex/Gender	0.36	0.57
	Prematurity	0.04	−0.47
	Low Birth Weight	0.13	0.52
	Ear Infection	0.30	0.45
	Visual or hearing impairment	0.10	0.24
	Family history	−0.01	−0.22
	Maternal education	−0.38	−0.12
	Household income	0.66	0.46
	Concern at time 1	0.08	0.17
	Productive vocabulary group	−0.62	−0.08
	Receptive vocabulary group	0.09	0.10
	Gesture group	0.09	−0.13

The second discriminant function analysis tested only the effect of health risk factors at time 1: child sex/gender, prematurity, low birth weight, ear infections, familial risk for speech or language impairment, developmental disability, and visual or hearing impairments. This model correctly classified children into their groups with an accuracy of 86.80%, *r* = 0.35, *χ*^2^ = 1790, *df* = 6, *p* = 0.006. Again, although the specificity of the complete model was excellent (98.40%) meaning it correctly classified almost all children in the No Identified Disability group Disability, it did not do well in terms of correctly classifying children in the Identified Disability group (sensitivity = 10.50%).

The third discriminant function analysis tested only the effect of demographic factors: maternal education and household income. This model failed to correctly classified children into their groups, *r* = 0.16, *χ*^2^ = 3.26, *df* = 2, *p* = 0.16. Again, the accuracy of the model was good (86.30%), and the sensitivity was high (100.00%), sensitivity was poor (0.00%).

The fourth discriminant function analysis tested only the effect of parental concern for language development at time 1. This model failed to correctly classify children into their groups, with an accuracy of 86.30%, *r* = 0.13, *χ*^2^ = 2.39, *df* = 1, *p* = 0.12. Again, the sensitivity of this model was poor, 0.00%, therefore it did not do well in terms of correctly classifying children with an identified disability at time 2. The specificity of the model, however, was excellent (100.00%) as it correctly classified all children without an identified disability at time 2.

The fifth discriminant function analysis included all risk factors and language group at time 1 to determine if these variables together are better predictors of group membership than separately. The included risk factors were health factors (child sex/gender, prematurity, low birth weight, ear infections at time 1, familial risk for speech or language impairment, developmental disability, visual or hearing impairment), demographic factors (maternal education, household income), concern expressed at time 1 (hearing or communication concerns at time 1), and language groups at time 1 (quartile groups for productive and receptive vocabulary and for gestures). This model correctly classified children into these two groups with an accuracy rate of 86.10%, *r* = 0.40, χ^2^ = 22.87, *df* = 12, *p* = 0.03. Again, however, as with all the previous models, though specificity was good (96.80%), sensitivity was poor (15.80%). In sum, all of the models had low sensitivity, and were thus unable to identify children in the Identified Disability group with reliable levels of accuracy.

### 3.3. Predicting overall concern for language at time 2 from risk factors and language at time 1

Since many children do not have a diagnosis of a language-related disorder by the time they are 4–6 years old, even if one is present, we ran these five analyses again with the broader category that included children with both an identified disability and children for whom a parent or professional had expressed ‘concern’ about their language development. For these analyses, children were split into two groups: Overall Concern and No Overall Concern. Here the sensitivity value measures the ability of the model to correctly classify children in the Overall Concern group (true positives) and specificity measure the model to correctly classify children in the No Overall Concern group (true negatives). The results from these five analyses can be seen in Tables 5, 6. As with the previous five analyses, the sensitivity of these models was very poor; they were unable to accurately classify children for whom there was concern about their language development.

**Table 6:** Results from the discriminant function analyses distinguishing between children with and without overall concern for their language development at time 2.

Variable	*r*	*χ*2	*n*	df	*p*	Accuracy	Sensitivity	Specificity
Language group at time 1 (quartile groups for vocabulary and gesture scores at time 1)	0.35	18.89	146	3	<0.001	67.10%	40.80%	80.40%
Health factors (sex/gender, prematurity, low birth weight, ear infection, visual or hearing impairment, family history, developmental disability)	0.33	15.40	143	6	0.02	70.80%	14.90%	97.90%
Demographic factors (maternal education, family income)	0.25	9.48	146	2	0.009	71.20%	34.70%	89.70%
Concern at time 1	0.15	3.35	146	1	0.07	67.80%	12.20%	95.90%
All variables	0.50	39.26	143	12	<0.001	75.70%	42.60%	91.80%

### 3.4. Predicting catch-up in language development from risk factors recorded at time 1

In this section of analysis we tested whether our risk factors and language scores at time 1, when combined, could predict ‘catch up ability’ (i.e., could distinguish between children whose language had been of concern at some point in their development but whose difficulties resolved by time 2, and those whose language was still of concern). This analysis included only the subset of children whose parents answered yes to the question “Have you ever worried that your child’s speech was delayed compared to other children the same age?” at time 2. For this analysis, the independent variables were all of the risk factors (child sex/gender, prematurity, low birth weight, ear infections at time 1, familial risk for speech of language impairment, developmental disability, visual or hearing impairment, maternal education, household income, concern at time 1), as well as vocabulary and gesture scores at time 1. The dependent variable was the answers to the language catch-up question at time 2 (“Did your child’s speech eventually catch up to that of other children the same age?”). A total of 37 parents expressed concern for their children’s language development at time 2. Of these 37, 26 reported that their children’s language had caught up with children the same age, and 11 reported that it had not caught up.

We ran a discriminant function analysis to assess the discriminatory ability of our time 1 risk/language factors to correctly classify children whose language did (0) and did not (1) catch up by time 2. This model did not correctly classify children into their groups. While the accuracy of this model was good, 82.90%, it did not reach significance, *r* = 0.57, χ^2^ = 10.66, *df* = 11, *p* = 0.47. This result is reflected in the sensitivity of the model. The sensitivity of this model was poor, 54.50% meaning it did not do well at classifying children whose language did not catch up. The specificity of the model was good, 95.80%, meaning it did well in terms of correctly classifying children whose language did catch up. See Table 7 for the results of the discriminant function analysis and Table 8 for the standardized canonical coefficients for each variable.

**Table 7:** Results of the discriminant function analysis distinguishing between children whose language did and did not catch up.

Variable	*r*	*χ*2	*n*	df	*p*	Accuracy	Sensitivity	Specificity
All variables	0.57	10.66	35	11	0.47	82.90%	54.50%	95.80%

Only 35 children (of the 37 whose language was of concern at some point) had data available for all variables in this analysis.

**Table 8:** Standardized canonical discriminant function coefficients for each variable predicting whether or not children’s language caught up after concern was expressed.

Model	Variable	r
All variables	Sex/Gender	−0.53
	Prematurity	0.69
	Low birth weight	−0.17
	Ear infection	−0.15
	Visual or hearing impairment	0.51
	Family history	0.18
	Maternal education	NA^!^
	Household income	−0.13
	Concern at time 1	−0.63
	Productive vocabulary group	−0.22
	Receptive vocabulary group	−0.38
	Gesture group	1.03

! Only one child whose language did or did not catch up had low maternal education so it did not contribute to classification accuracy.

### 3.5. Exploratory analyses

We ran three exploratory descriptive analyses to investigate possible reasons why the risk factors we identified were not sensitive enough to correctly classify children at time 2. We focussed on the concern measures, since few children had been diagnosed with a language-related disorder at time 2. Table 9 details the number and percentage of children in each Concern group (Overall Concern, No Overall Concern) with each risk factor. We can see from this table that the proportion of children with the risk factor is almost always bigger in the Overall Concern group than the No Overall Concern group. For example, if we consider the family history of language delay or dyslexia risk factor, 20.83% of children in the Overall Concern group have that risk factor, compared to 14.43% in the No Overall Concern group. Thus, the expected differences in the prevalence and number of risk factors between groups is present in our sample. However, the differences are not big; for most risk factors, a substantial minority of children in the No Overall Concern group also have the risk factor.

**Table 9:** Number and percentage of children with each risk factor split by Overall Concern and No Overall Concern groups.

Variable	Have risk factor	Overall Concern *n* (%)	No Overall Concern *n* (%)
Sex/Gender (male)	Yes	33(67.35%)	43(44.33%)
	No	16(32.65%)	54(55.67%)
Prematurity	Yes	5(10.20%)	8(8.25%)
	No	44(89.80%)	89(91.75%)
Low birth weight	Yes	5(10.20%)	6(6.19%)
	No	44(89.80%)	91(93.81%)
Ear infection at time 1	Yes	3(6.12%)	0(0.00%)
	No	46(93.88%)	97(100.00%)
Family history	Yes	10(20.83%)	14(14.43%)
	No	38(79.17%)	83(85.57%)
Developmental disability at time 1	Yes	0(0.00%)	0(0.00%)
	No	48(100.00%)	97(100.00%)
Visual impairment at time 1	Yes	1(2.13%)	1(1.03%)
	No	46(97.87%)	96(98.97%)
Concern at time 1	Yes	6(12.24%)	4(4.12%)
	No	43(87.76%)	93(95.88%)
Maternal education	Yes	3(6.12%)	9(9.28%)
	No	46(93.88%)	88(90.72%)
Family income	Yes	18(36.73%)	16(16.49%)
	No	31(63.27%)	81(83.51%)
Productive vocabulary at 15–18 months	0–25th percentile	20(40.82%)	19(19.59%)
	25–50th percentile	17(34.69%)	13(13.40%)
	50–75th percentile	6(12.24%)	33(34.02%)
	75th percentile and above	6(12.24%)	32(32.99%)
Receptive vocabulary at 15–18 months	0–25th percentile	14(28.57%)	19(19.59%)
	25–50th percentile	13(26.53%)	19(19.59%)
	50–75th percentile	12(24.49%)	20(20.62%)
	75th percentile and above	10(20.41%)	39(40.21%)
Gesture scores at 15–18 months	0–25th percentile	15(30.61%)	15(15.46%)
	25–50th percentile	13(26.53%)	23(23.71%)
	50–75th percentile	8(16.33%)	35(36.08%)
	75th percentile and above	13(26.53%)	24(24.74%)

Next, we looked at the number and proportion of children with and without overall concern in each of the language quartile groups at time 1 (Table 9). We can see from Table 9 why language and gesture scores at time 1 do not discriminate between groups at time 2. Again, although there are a greater proportion of children in the lowest quartiles who subsequently raise concerns than in the higher quartiles (e.g., 40.82% for 0–25th percentile vs. 12.24% in 75–100th percentile for productive vocabulary) the differences are not large or distinct enough to be discriminatory. A substantial minority of children in the higher quartiles go on to develop language that is of concern, and a substantial minority of children in the lower quartiles do not.

Finally, we created total risk factor scores for each child in the Overall Concern and No Overall Concern groups. The total number of risk factors was 10 (being a boy, being premature, having a low birth weight, ear infections, family history of language delay or dyslexia, having a developmental disability at time 1, having a visual or hearing impairment at time 1, hearing or communication concerns at time 1, low maternal education and low household income). The mean number of risk factors for children in the Overall Concern group was 1.71 (SD = 1.14) and the mean number of risk factors for children in the No Overall Concern group was 1.04 (SD = 1.09). Thus, once again, although the difference was in the expected direction, it was not large. In addition, the overlap in standard deviation of both groups was big, and the range was the same across the two groups (0–5). Therefore, while children in the Overall Concern group experience a larger number of risk factors overall, the differences are almost certainly not big or distinct enough to be discriminatory.

## 4. Discussion

The goal of the present study was to investigate if we could use a combination of risk factors, earlier vocabulary and gesture scores, as well as early parental concern about language at time 1 to predict (a) which children will have an identified language disability and (b) later concern for language development at time 2.

We used discriminant function analyses to examine if different combinations of health and demographic risk factors as well as earlier vocabulary and gesture scores, and concern measures, could discriminate children who had, at 4–6 years (time 2), either an identified language-related disability (Identified Disability) or for whom concern about language development had been registered by a parent or professional (Overall Concern). None of our models yielded successful predictors to identify either disability or concern.

We first examined the role of earlier language and gesture scores but these variables did not successfully discriminate between children (i.e., failed to identify both children with an identified disability and children in the Overall Concern groups). When we examine the number of children in each vocabulary and gesture quartile (Table 9), we see why: children in the two groups were distributed across all four quartiles with very little clustering at each end for each group. For example, only 19.59% and 13.40% of children in the overall Concern group at time 2 were in the bottom 0–25th and 25–50th, respectively, at time 1.

We then examined a number of health or demographic risk factors. As with early language skills above, these risk factors did not successfully identify children who had an identified disability or who were in the Overall Concern group. Again, our exploratory analyses shows why this was the case. For example, although the children in the Overall Concern group were reported at time 1 to have experienced a greater number of risk factors on average (1.71 vs. 1.04), there was considerable overlap (wide and overlapping standard deviations and ranges) in the number of risk factors in the Overall Concern/No Overall Concern groups. This means that no combination of health or demographic risk factors was discriminant enough to distinguish between these two groups of children. Furthermore, when we consider that there were a maximum of 10 risk factors and the most risk factors any one child experienced was 5, we can see that neither children with, nor children without, overall concern for their language development were exposed to a very high number of these risk factors. This result is consistent with previous research, which has shown that health and demographic risk factors are better at predicting individual differences than they are at predicting language impairment or concern for language development (Harrison and McLeod, 2010; Reilly et al., 2010). For example, Harrison and McLeod (2010) found health and demographic risk factors did not predict parental concern for vocabulary development or use of speech-language pathology services, with even a combination of these factors yielding poor levels of sensitivity.

Even our full model, which included all risk factors, earlier vocabulary and gesture scores and early concern for language development at time 1, failed to identify individual children who had an identified disability or who were in the Overall Concern group at time 2. This may seem surprising given the wealth of evidence suggesting, for example, that some children who are late talkers in early childhood are more at risk of later language delay (Bishop and Adams, 1990; Rescorla, 2002). However, our findings are in line with much of the previous research that focusses on predicting *which* late talkers will develop language disorder. For example, Reilly et al. (2010) found that while risk factors were successful in predicting continuous language scores at 4 years (i.e., individual differences), they were unable to correctly classify children with DLD. Our findings are also in line with a recent systematic review and meta-analysis of screening tools for language disorder, which concluded that only a very small number (13.8%) of the 67 screening tools tested yielded good accuracy at identifying children with language disorder (So and To, 2022).

We then examined the stability of parental concern over time. Again, contrary to our predictions, overall concern at time 2 was not predicted by parental concern at time 1. Previous research has shown that parental concern for language delay can benefit clinical detection of language impairment (Glascoe, 1991; Glascoe and Dworkin, 1995), but this research has typically been carried out with older children. The findings here suggest that early concern for language development may not be as beneficial for predicting later problems. However, it is important to note that very few parents reported concern at time 1; only 6 parents of children who expressed concern at time 2 also expressed concern at time 1 (see Table 9). Therefore, we are hesitant to draw a firm conclusion that there is no relationship between parental concern at time 1 and Overall Concern at time 2.

Finally, we investigated if catch-up in language delay is associated with exposure to fewer risk factors and better scores on earlier vocabulary and gestures. In line with the results of the previous models, this model had poor sensitivity and did not correctly classify children whose language did not catch up (i.e., children whose language development was of the greatest concern).

One could argue that one reason for our overall pattern of results could be the narrowness of our sample in terms of demographic characteristics. Although the full UK-CDI sample was representative of the UK population, we found that parents from families with higher income and higher maternal education were more likely to respond to the follow-up questionnaire (see Supplementary materials https://osf.io/gvz3x/). This is a common problem when trying to collect representative samples (Reilly et al., 2010). However, we do not think that this explains our pattern of results. Just under 14% of our follow up sample had an identified disability, compared to 7.58% of all children in the UK population with DLD (Norbury et al., 2016). Thus, we would argue that we have data from a representative enough portion of the population, at least when it comes to the incidence of language-related disability, if not socio-economic status. In addition, there were no differences in the sample characteristics of those who did/did not take part in the follow up study in terms of the other risk factors. That said, it is important that low SES families are represented in studies, so future research should therefore make an increased effort to contact families represented in lower SES brackets. We may have been more successful at encouraging families to participate if we had personally contacted them, either *via* email or phone.

Another possible reason for the lack of predictiveness at the individual level could lie in children’s changing environments between 15 and 18 months and 4–6 years having a substantial effect on their language development. Previous research has shown that, once children start attending playgroups and nurseries (typically at about 1–2 years of age in the UK), this has a substantial impact on their cognitive development (Turner, 1974; Sylva et al., 2011). Hence, it is plausible that these differences also impact on language development. The growing influence of peers and nursery staff on children contributes to their language diversification and growth, as well as to vocabulary growth and complexity of language. For example, children who hear little language in the home, and may develop slowly at first, might start to be looked after by grandparents or attend a nursery that promotes language development, and thus start to enhance their productive and receptive vocabulary and grammar. In other words, changes in children’s environment over the preschool years may weaken the link between early and later language development.

Given that the profile of children with persisting language impairments into later childhood tends to be characterized by greater difficulties in syntax or pragmatics (Norbury and Bishop, 2002; Bishop, 2014) than in vocabulary (Bishop and Snowling, 2004; Leonard, 2014), it is worth to pay attention to underlying mechanisms. It is possible that the cognitive mechanisms that underpin language acquisition early in life (where the focus is on learning to use and interpret gestures and words) are not at the root of language impairment later in childhood (where development is more focussed on syntax and pragmatics). While some (e.g., Locke, 1997) propose that the development of lexicon and innate syntactic complexity emerge from separate linguistic mechanisms that activate in sequence, others suggest that more fundamental learning abilities including categorization and pattern recognition, joint attention, intention-reading and input analysis are emergent, and such joint factors drive the development of vocabulary and of grammatical constructions over time (e.g., De Ruiter et al., 2020). On both above approaches, there might be a different and changing relationship between vocabulary and syntax, but not one that is strong enough to predict later concern for language development. Alternatively, it may be possible that different (but partially overlapping) mechanisms underlie vocabulary and syntax acquisition, and, thus, that delays in vocabulary and syntax acquisition stem from different causes (see van der Lely et al., 1998, for a theory of specific language impairment based on this premise).

In line with this, one possible method for identifying which children go on to have persisting language impairments would be to examine the composition of late talker’s vocabulary (Perry et al., 2022). Perry et al. (2022) found that late talking children produced significantly fewer shape-based nouns compared to typically developing children. It is possible that in addition to grouping children by quartiles, greater accuracy in predicting later language-related disabilities could be achieved by analyzing the composition of children’s vocabulary when they are between 15 and 18 months old. However, this method would be limited to children who are already producing words at this age.

Furthermore, a shift in parental perception may also explain the independence of parental concern measures at time 1 and time 2; it is possible that the factors driving parental concern at time 1 and time 2 are not the same. For example, when children are 15–18 months old, a delay in productive vocabulary development is likely to be responsible for parental concern. However, by the time children are 4–6 years old, concern may focus on difficulties associated with syntax acquisition (Leonard, 1998; Bishop and Snowling, 2004; Leonard, 2014). Therefore, parental concern at time 1 and time 2 may stem from different sources.

One potential limitation is the use of parental report of developmental disability and the effect of recall bias. Relying on parental reports may not provide a completely accurate picture of their earlier concern for their children’s language development. It is possible that a subsequent diagnosis of a developmental disability may result in parents misremembering their concern for their children’s language development at an earlier timepoint. However, our analyses predicting Identified Disability controlled for any inflation in parental report of concern for language development. In addition, if there was an inflation in parental concern for language development due to a diagnosis of a developmental disability, it did not impact our ability to discriminate between children with and without concern for their language development.

In sum, throughout all of our analyses, there was an interesting overall picture: While we can predict reliably which children will go on to develop in a typical fashion, we fail to predict individual children with an identified disability, or with an overall concern for their language development. Given the number of robust correlations between early and later language, and between risk factor variables and language development in the literature, this may seem like a surprising result. However, our research has shown that such associations are not precise or accurate enough to enable us to identify individual children at risk of language disability.

## 5. Conclusion

The present findings shed light on the role of early language delays, and of health and demographic risk factors, in predicting later language outcomes. Such risk factors are currently recommended as a starting point when monitoring language delay in young children because the incidence of persisting language impairments is greater in children from families exposed to greater risks (Harrison and McLeod, 2010). However, we have found that these risk factors, either alone or when combined with early language measures, do not allow us to accurately predict identified disability or concern for children’s language development over time. Thus, we cannot currently recommend that they be used as described above to screen individual children at risk for language impairment and further research needs to be carried out improve sensitivity.

## Data availability statement

The raw data supporting the conclusions of this article will be made available by the authors, without undue reservation.

## Ethics statement

The studies involving human participants were reviewed and approved by The University of Liverpool’s Research Ethics Subcommittee for Non-Invasive Procedures (Institute Review Board protocol number: RETH000764). Written informed consent to participate in this study was provided by the participants’ legal guardian/next of kin.

## Author contributions

LJ and CR designed the follow-up study. CR, KA, and KM ran the original UK-CDI Project and contributed to the development of the manuscript. CR and KA contributed to the design of the follow-up questionnaire. JP contributed to the development of the manuscript. All authors contributed to the article and approved the submitted version.

## Funding

This study was completed as part of the first author’s PhD. The funding for this PhD came from the ESRC International Centre for Language and Communicative Development [ES/L008955/1].

## Conflict of interest

The authors declare that the research was conducted in the absence of any commercial or financial relationships that could be construed as a potential conflict of interest.

## Editor’s note

Maria-José Ezeizabarrena edited the article in collaboration with Melita Kovacevic, University of Zagreb, Zagreb, Croatia.

## Publisher’s note

All claims expressed in this article are solely those of the authors and do not necessarily represent those of their affiliated organizations, or those of the publisher, the editors and the reviewers. Any product that may be evaluated in this article, or claim that may be made by its manufacturer, is not guaranteed or endorsed by the publisher.

## Footnotes

^1^We use the term sex/gender to indicate that a child’s development can be affected both by their biological sex and socially-constructed gender. Although children at the age studied in this paper are unlikely to have already chosen their preferred gender, their development is very likely to have been influenced by the gender that their parents/society has assigned to them, as well as their biological sex. Thus we use the term sex/gender to encompass both these effects (see Bölte et al., 2023).
