# Erratum: Language outcomes from the UK-CDI Project: can risk factors, vocabulary skills and gesture scores in infancy predict later language disorders or concern for language development?

**DOI:** 10.3389/fpsyg.2023.1258720

**Published:** 2023-08-09

**Authors:** Frontiers Production Office

**Affiliations:** Frontiers Media SA, Lausanne, Switzerland

**Keywords:** vocabulary, CDI, health risk factors, demographic risk factors, language development, language impairment, language disorder

Due to a publishing error, co-Editor accreditation needs to be amended. An Editor's Note has been added to the original article, as shown below.

**Editor's note**

Maria-José Ezeizabarrena edited the article in collaboration with Melita Kovacevic, University of Zagreb, Zagreb, Croatia.

The publisher apologizes for this mistake. The original article has been updated.

