# Supplementary material for: Language outcomes from the UK-CDI Project: can risk factors, vocabulary skills and gesture scores in infancy predict later language disorders or concern for language development?
Source: Front Psychol. 2023 Jun 16;14:1167810. doi: 10.3389/fpsyg.2023.1167810 (PMC10313203; doi:10.3389/fpsyg.2023.1167810)
Supplement: Supplementary file 1 [file Data_Sheet_1.pdf]

UNIVERSITY OF LIVERPOOL

UK-CDI Follow Up Questionnaire

Background Information

Please provide the following information

1. Your first name

---

2. Your surname

---

3. Child’s first name

---

4. Child’s surname

---

5. Address line one

---

6. City

---

7. Postcode

---

8. Your email address

---

9. Your relationship to child

---

10. Today’s Date (please provide the date you filled out the questionnaire)

---

11. What is your child's date of birth?

---

12. Is your child male or female?

☐ Male ☐ Female

Your Child's Development

13. Have you ever worried that your child's speech was delayed compared to other children the same age?

☐ Yes
☐ No
If you answered **No** to question 13, please skip to question 17

14. Did your child's speech eventually catch up with that of other children the same age?

☐ Yes
☐ No
If you wish, you can provide more information here

15. Around what age did you notice your child's language catching up? If possible, try to remember how old he or she was in years and months

___

If you wish, you can also include more information in the below

___

___

16. Did you seek any additional support to aid your child's language development? For example, did you talk to a health visitor, was your children referred to Speech and Language Therapy?

If yes, please provide details

☐ Yes ___
☐ No
If you wish, you can provide more information here

___

17. Does your child have a developmental disability (e.g. Cerebral Palsy, Autism Spectrum Disorder (ASD), Fragile X syndrome, Muscular dystrophy, Di George syndrome, Down syndrome, Williams syndrome)?

If yes, please provide details

☐ Yes ___
☐ No
If you wish, you can provide more information here

___

18. Has your child been diagnosed with any of the following language disorders?

If other, please provide details

☐ Specific Language Impairment/Developmental Language Disorder
☐ Dyslexia
☐ Other ____
☐ No
If you wish, you can provide more information here

____

19. Does your child have a hearing or visual impairment?

If yes, please provide details

☐ Yes ____
☐ No
If you wish, you can provide more information here

____

20. Does your child regularly hear another language that is not English?

☐ Yes
☐ No
If you wish, you can provide more information here

____

If you answered **No** to question 20, please skip to question 23

21. What is this language?

____

22. For how many hours does your child hear this other language in a typical week?

____

23. Does your child live at another home for part of the year?

☐ Yes
☐ No
If you wish, you can provide more information here

____

If you answered **No** to question 23, please skip to question 26

24. Please give the postcode of this other home

____

25. How long do they spend at this other home?

☐ Less than half the year
☐ About half the year
☐ More than half the year

26. Were you and your child invited to take part in the Healthy Child Programme's 2 Year Review, also known as the Two Year Check

The Healthy Child Programme is a public health programme run by Public Health England. You will have been invited to the review by your Health Visitor

☐ Yes
☐ No
If you wish, you can provide more information here

____

If you answered **No** to question 26, please skip to question 29

27. Did this programme identify any delays with your child's speech, language or communication abilities?

☐ Yes
☐ No
If you wish, you can provide more information here

____

28. Were you given any guidance to help boost these areas of development?

☐ Yes
☐ No
If you wish, you can provide more information here

____

Your Child's Family

29. Is there anyone in the child’s immediate family (brothers/sisters/parents only) with a speech or language difficulty or dyslexia?

☐ Yes ____
☐ No

If you wish, you can provide more information here

---

30. Does your child have any older siblings (brothers or sisters)?

☐ Yes
☐ No
If you answered **No** to question 30, please skip to question 32

31. How many older siblings do they have? (Include full or half siblings)

☐ 1 ☐ 2 ☐ 3 or more

32. Does your child have any younger siblings?

☐ Yes
☐ No
If you answered **No** to question 32, please skip to question 34

33. How many younger siblings do they have?

☐ 1 ☐ 2 ☐ 3 or more

Your Child's Mum

34. Mum is…

☐ Single ☐ Married/Civil Partnered ☐ Living with Partner

☐ Separated /Divorced ☐ Widowed

35. Mum's highest education is

☐ No formal qualifications

☐ GCSE/O Level /NVQ Level 1 or 2/ similar

☐ A Level/NVQ Level 3/ similar

☐ University degree/HND/HNC/NVQ Level 4 or 5/similar

☐ Postgraduate/similar e.g. (PGCE, PhD, MA etc.)

36. Mum's work status is

☐ Never worked, have only been in training or education

☐ Unemployed

☐ An employee

☐ Self-employed (with employees)

☐ Self-employed (without employees)

☐ Student

If you answered **An employee** or **Self-employed (with employees)** please indicate how many people work for Mum’s employer or for Mum if she is/was an employer?

☐ 0
☐ 1-24
☐ 25+

37. Mum's current or last job title (please be specific):

____

____

Your Child's Dad

38. Dad is...

☐ Single ☐ Married/Civil Partnered ☐ Living with Partner

☐ Separated /Divorced ☐ Widowed

39. Dad's highest education is

☐ No formal qualifications

☐ GCSE/O Level /NVQ Level 1 or 2/ similar

☐ A Level/NVQ Level 3/ similar

☐ University degree/HND/HNC/NVQ Level 4 or 5/similar

☐ Postgraduate/similar e.g. (PGCE, PhD, MA etc.)

40. Dad's work status is

☐ Never worked, have only been in training or education

☐ Unemployed

☐ An employee

☐ Self-employed (with employees)

☐ Self-employed (without employees)

☐ Student

If you answered **An employee** or **Self-employed (with employees)** please indicate how many people work for Dad's employer or for Dad if he is/was an employer?

☐ 0
☐ 1-24
☐ 25+

41. Dad's current or last job title (please be specific):

____

____

Family Background

42. What is the overall household income (before tax) per year in your child's main home?

☐ £0-£14,000

☐ £14,001-£24,000

☐ £24,001-£42,000

☐ £42,001 or more

☐ Prefer not to say

Your Child's Day

43. Who looks after your child? Please tell us about everyone who looks after your child for at least half a day in a typical week.

☐ Child's Mum

☐ Child's Dad

☐ Other carer or carers

☐ At school

If you answered **Other carers or carer** to question 43 please answer question 44 & 45

44. Other carers are:

☐ Family

☐ Childminder

☐ Nursery

If you wish, you can provide more information here

____

45. How many hours do these other carers look after your child in a typical week?

☐ 1-20 hours

☐ 21-35 hours

☐ 36+ hours

If you answered **At schoo**l to question 43 please answer question 46

46. After school, who looks after your child? Select all options that apply

☐ Mum and/or Dad

☐ Another family member

☐ A childminder

☐ An after school club

☐ Other (please specify) ____

47. How many hours per day- including nap time- does your child sleep?

☐ Less than 9 hours

☐ 9 hours

☐ 10 hours

☐ 11 hours

☐ 12 hours

☐ 13 hours

☐ 14 hours

☐ 15+ hours

Please provide your email address below so we can send you the shopping voucher

____

Date: ……………………….
