# Supplementary material for: Language outcomes from the UK-CDI Project: can risk factors, vocabulary skills and gesture scores in infancy predict later language disorders or concern for language development?
Source: Front Psychol. 2023 Jun 16;14:1167810. doi: 10.3389/fpsyg.2023.1167810 (PMC10313203; doi:10.3389/fpsyg.2023.1167810)
Supplement: Supplementary file 2 [file Table_1.docx]

Supplementary Materials

Language outcomes from the UK-CDI Project: can risk factors, vocabulary skills and gesture scores in infancy predict later concern for language development?

## Preliminary analysis: Demographics of families who did and did not reply to the follow-up questionnaire

We ran Chi^2^ analyses to investigate if there were any differences in the risk factors listed in table S1 in the supplementary materials, recorded at time 1, between families who did and did not respond to the follow-up questionnaire. There was only a significant difference between these two groups in terms of in maternal education (χ^2^ (4)=23.25, *p*=.0001) and household income (χ^2^ (3)=22.03, *p*=.0002). There were no significant differences between the groups in terms of prematurity, birth weight, incidence of ear infections, family history of language delay or dyslexia, developmental disability, visual or hearing impairment and hearing or communication concerns. The results for these analyses, along with percentages of children in each response group who had each risk factor at time 1, can be seen in table S1.

Table S1

*Number and percentage of children in each risk factor category whose parents did and did not reply. Results are split by sex . The Chi^2^ analysis is a comparison of all families who did and did not reply, not split by sex*

|  | | Female | | Male | |  |  |  |
| --- | --- | --- | --- | --- | --- | --- | --- | --- |
| Risk factor | | Families who replied (%) | Families who did not reply (%) | Families who replied (%) | Families who did not reply (%) | χ^2^ | df | p |
| Health problems | |  |  |  |  |  |  |  |
|  | Prematurity time 1 | 5 (7%) | 6 (5%) | 8 (11%) | 11 (10%) | 1.86 | 2 | .39 |
|  | Low birth weight time 1 | 6 (9%) | 8 (7%) | 5 (7%) | 5 (5%) | 0.77 | 2 | .68 |
|  | Ear infection at time 1 | 1 (1%) | 1 (1%) | 2 (3%) | 7 (6%) | 0.73 | 1 | .39 |
|  | Familial risk time 1 | 12 (17%) | 17 (15%) | 12 (16%) | 19 (17%) | 0.01 | 1 | .92 |
|  | Developmental disability time 1 | 5 (7%) | 6 (5%) | 8 (11%) | 11 (10%) | 0.67 | 1 | .41 |
|  | Visual or hearing impairment | 6 (9%) | 8 (7%) | 5 (7%) | 5 (5%) | 0.71 | 1 | .40 |
| Language concerns at time 1 | |  |  |  |  |  |  |  |
|  | Hearing or communication concerns at time 1 | 1 (1%) | 1 (1%) | 1 (1%) | 5 (5%) | 0.04 | 1 | .85 |
| Demographic factors | |  |  |  |  |  |  |  |
|  | Maternal education time 1 | 2 (3%) | 4 (4%) | 8 (11%) | 10 (9%) | 23.25 | 4 | .0001 |
|  | Household income time 1 | 2 (3%) | 4 (4%) | 8 (11%) | 10 (9%) | 22.03 | 3 | .0002 |
